# Tailoring Surface Frustrated Lewis Pairs of In_2_O_3−_ *_x_*(OH)_y_ for Gas‐Phase Heterogeneous Photocatalytic Reduction of CO_2_ by Isomorphous Substitution of In^3+^ with Bi^3+^

**DOI:** 10.1002/advs.201700732

**Published:** 2018-03-12

**Authors:** Yuchan Dong, Kulbir Kaur Ghuman, Radian Popescu, Paul N. Duchesne, Wenjie Zhou, Joel Y. Y. Loh, Abdinoor A. Jelle, Jia Jia, Di Wang, Xiaoke Mu, Christian Kübel, Lu Wang, Le He, Mireille Ghoussoub, Qiang Wang, Thomas E. Wood, Laura M. Reyes, Peng Zhang, Nazir P. Kherani, Chandra Veer Singh, Geoffrey A. Ozin

**Affiliations:** ^1^ Department of Chemistry University of Toronto 80 St. George Street, Rm 326 Toronto Ontario M5S 3H6 Canada; ^2^ Department of Materials Science and Engineering University of Toronto 184 College Street, Suite 140 Toronto Ontario M5S 3E4 Canada; ^3^ Laboratory for Electron Microscopy (LEM) Karlsruhe Institute of Technology (KIT) Engesserstr. 7 76131 Karlsruhe Germany; ^4^ Department of Chemistry Dalhousie University 6274 Coburg Road, P.O. Box 15000 Halifax B3H 4R2 Canada; ^5^ The Edward S. Rogers Sr. Department of Electrical and Computer Engineering University of Toronto 10 King's College Road Toronto Ontario M5S 3G4 Canada; ^6^ Institute of Nanotechnology and Karlsruhe Nano Micro Facility Karlsruhe Institute of Technology Hermann‐von‐Helmholtz Platz 1 76344 Eggenstein‐Leopoldshafen Germany; ^7^ Helmholtz‐Institute Ulm for Electrochemical Energy Storage (HIU) Karlsruhe Institute of Technology (KIT) 89081 Ulm Germany; ^8^ Institute of Functional Nano and Soft Materials (FUNSOM) Soochow University Suzhou 215123 Jiangsu China; ^9^ Institute of Coal Chemistry Chinese Academy of Science 27 Taoyuan South Road Taiyuan 030001 Shanxi China

**Keywords:** carbon dioxide, isomorphous substitution, photocatalysts, solar fuels, surface‐frustrated Lewis pairs

## Abstract

Frustrated Lewis pairs (FLPs) created by sterically hindered Lewis acids and Lewis bases have shown their capacity for capturing and reacting with a variety of small molecules, including H_2_ and CO_2_, and thereby creating a new strategy for CO_2_ reduction. Here, the photocatalytic CO_2_ reduction behavior of defect‐laden indium oxide (In_2_O_3−_ *_x_*(OH)*_y_*) is greatly enhanced through isomorphous substitution of In^3+^ with Bi^3+^, providing fundamental insights into the catalytically active surface FLPs (i.e., In—OH···In) and the experimentally observed “volcano” relationship between the CO production rate and Bi^3+^ substitution level. According to density functional theory calculations at the optimal Bi^3+^ substitution level, the 6s^2^ electron pair of Bi^3+^ hybridizes with the oxygen in the neighboring In—OH Lewis base site, leading to mildly increased Lewis basicity without influencing the Lewis acidity of the nearby In Lewis acid site. Meanwhile, Bi^3+^ can act as an extra acid site, serving to maximize the heterolytic splitting of reactant H_2_, and results in a more hydridic hydride for more efficient CO_2_ reduction. This study demonstrates that isomorphous substitution can effectively optimize the reactivity of surface catalytic active sites in addition to influencing optoelectronic properties, affording a better understanding of the photocatalytic CO_2_ reduction mechanism.

## 1 Introduction

Photocatalytic reduction of carbon dioxide (CO_2_) has been explored for many years as an attractive strategy for reducing CO_2_ emissions while producing renewable fuels and chemicals.^1^, ^2^, ^3^ Oxide semiconductors are widely used as CO_2_ reduction photocatalysts due to their light‐harvesting property, high stability, and low cost.^4^, ^5^, ^6^, ^7^ However, due to the high thermodynamic stability of CO_2_ and the limitations of intrinsic semiconductor photocatalysts, such as poor charge separation and weak interaction with reactants, intensive efforts have been made to improve these oxide semiconductor photocatalysts via doping.^8^, ^9^ For instance, in fluorinated anatase TiO_2_ nanosheets prepared via substitution of surface hydroxyl groups with fluoride anions, surface fluorination promoted the formation of Ti^3+^ defects that helped extend the lifetime of photogenerated electrons and holes, and facilitated the reduction of CO_2_ to CO_2_^−^.^10^ In another study, copper‐loaded TiO_2_ catalysts prepared via a sol‐gel method showed a great increase in methanol production rate relative to nondoped TiO_2_. In this case, Cu^+^ also inhibits the recombination of photoexcited charge carriers without affecting electron mobility.^11^ Despite the large number of doped oxide semiconductor photocatalyst studies, the focus has been mostly on photoelectrochemical CO_2_ reduction, and explanations for the improved catalytic activity are still mainly limited to optoelectronic properties such as bandgap, light absorption efficiency, charge carrier lifetimes, and electron mobility.^12^, ^13^, ^14^, ^15^ However, like other catalytic reactions, the efficiency of photocatalytic CO_2_ reduction relies heavily on the nature of catalytic sites at the photocatalyst surface, where the adsorption, activation, and reaction of gaseous reactants, and desorption of products, occur.^16^, ^17^, ^18^, ^19^, ^20^ Thus, it is important to examine how doping affects the surface chemistry and catalytic process in order to rationally design catalytic materials capable of replacing nonrenewable fossil fuels with renewable synthetic fuels through photocatalytic CO_2_ reduction.

Gas‐phase photocatalytic conversion of CO_2_ to CO via the endothermic reverse water gas shift (RWGS) reaction is promising both from fundamental and practical points view, as the CO produced can be utilized directly as the feedstock for producing chemicals and fuels (e.g., via the Fischer–Tropsch process).^21^ Herein, we demonstrate an optimal level of Bi^3+^ doping into the lattice of In_2_O_3−_ *_x_*(OH)*_y_* nanocrystals, thereby achieving a remarkable enhancement of RWGS reactivity that can be ascribed to optimization of the Lewis acidity and Lewis basicity of surface frustrated Lewis pairs (FLPs). In the molecular FLPs system, originally developed by Stephan and Erker, a Lewis acid and Lewis base are sterically prevented from bond formation, and can thus act cooperatively to capture and react with a variety of small molecules, such as during the heterolytic dissociation of H_2_ and reaction with CO_2_.^22^, ^23^ Our recent studies have discovered FLPs on the surface of In_2_O_3−_ *_x_*(OH)*_y_* nanocrystals, with indium hydroxide groups as the Lewis base and coordinatively unsaturated indium sites as Lewis acid. Herein, these surface FLPs are denoted as In—OH···In. Detailed experimental and computational studies of the RWGS reaction process on In—OH···In FLPs demonstrated that H_2_ was first heterolytically dissociated into hydridic In—H and protonic O—H surface sites, forming In—OH_2_^+^···In—H^−^. Next, CO_2_ was activated and dissociated to CO with the concomitant formation of H_2_O.^24^, ^25^, ^26^ Inspired by the molecular FLP system, whose catalytic property can be tuned through elemental variation of the Lewis acid or Lewis base sites, the catalytic activity of In_2_O_3−_ *_x_*(OH)*_y_* in this study was optimized through isomorphous substitution of In^3+^ with Bi^3+^. The insight gained from density functional theory (DFT) calculations on this system provides compelling evidence that the In—OH···In FLP can be systematically tuned in order to maximize its catalytic activity toward heterogeneous CO_2_ reduction.

## 2 Results and Discussion

### 2.1 Structural and Surface Characterization

**Figure 1:**
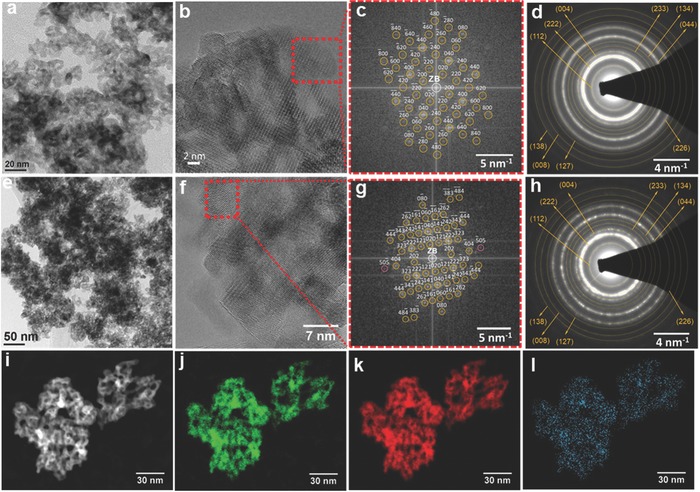
a,e) TEM overview images of nanoparticles within the Bi‐0% and Bi‐0.1% sample, respectively. b,f) HRTEM images of nanoparticle ensembles of the Bi‐0% and Bi‐0.1% sample, respectively; each dashed red frame encompasses a single monocrystalline nanoparticle. c) Experimental Fourier transformation and calculated diffraction pattern of the Bi‐0% single nanoparticle shown in the red frame with Miller indices for the bulk cubic In_2_O_3_ structure in the [001]‐zone axis (yellow circles); g) experimental Fourier transformation and calculated diffraction pattern of the single nanoparticle shown in the red frame with Miller indices for the bulk cubic In_2_O_3_ structure in the [101]‐zone axis (yellow and pink circles); pink circles indicate peaks with structure factors of zero within the diffraction pattern of pure In_2_O_3_, which become visible via Bi substitution (see text); d,h) indexed SAED patterns of Bi‐0% and Bi‐0.1% nanoparticle ensembles, respectively. i) HAADF‐STEM image and EDXS elemental maps of j) O (O‐K_α1_, green), k) In (In‐L_α1_, red), and l) Bi (Bi‐M_α_, blue) for nanoparticles within the Bi‐0.5% sample are shown, illustrating the homogeneous distribution of O, In, and Bi.

**Figure 2:**
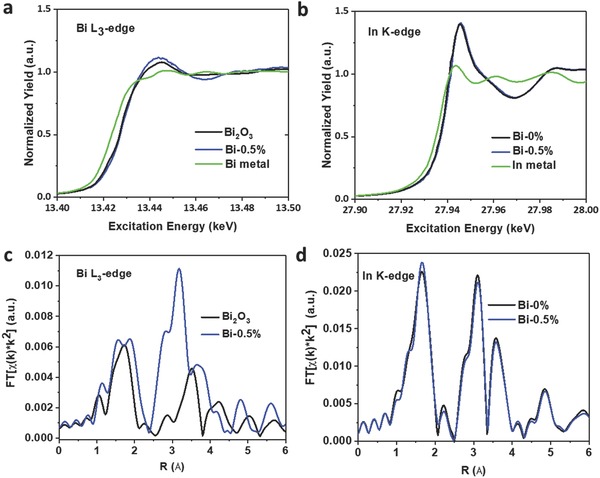
a,b) Normalized Bi L_3_‐edge and In K‐edge XANES spectra of Bi‐0.5%. c,d) Fourier‐transformed EXAFS spectra of Bi‐0.5% in comparison to unsubstituted In_2_O_3−_ *_x_*(OH)*_y_* (Bi‐0%) and reference Bi_2_O_3_.

In this work, a series of Bi^3+^‐substituted In_2_O_3−_ *_x_*OH*_y_* nanocrystals were prepared via dehydroxylation of Bi^3+^‐substituted In(OH)_3_ in air at 250 °C for 6 h. These samples are referred to as Bi‐0.03%, Bi‐0.05%, Bi‐0.08%, Bi‐0.1%, and Bi‐0.5 based on the nominal molar ratio of bismuth to indium in the synthesis, respectively. **Figure** **1**a–c,e–g depicts the transmission electron microscopy (TEM) images of Bi‐0% and Bi‐0.1%, respectively. High‐resolution TEM (HRTEM) images (Figure 1b,f) show lattice fringes which are continuously distributed over large areas, with pores between interconnected nanocrystals revealing their crystalline structure. The particle within the dashed frame of Figure 1b is a single In_2_O_3−_ *_x_*(OH)*_y_* monocrystal with a cubic structure, as indicated by the good agreement between its 2D Fourier transform and the calculated diffraction pattern of bulk cubic In_2_O_3_ (space group Ia‐3, space group number 206, and a lattice parameter of *a* = 10.146 Å) in the [001]‐zone axis (Figure 1c). Also, in the case of the Bi‐0.1% sample, the 2D Fourier transform of the particle within the dashed frame in Figure 1f was in good agreement with the calculated diffraction pattern of bulk cubic In_2_O_3_ in the [101]‐zone axis (Figure 1g). It is significant that, within pure cubic In_2_O_3_, the reflections indicated in pink should not be observed, because their structure factors are zero. They can only be observed if some In atoms are substituted by Bi atoms while maintaining the bulk cubic In_2_O_3_ structure. This provides strong support for the small doping level of In_2_O_3_ with Bi^3+^ and its uniform distribution into the lattice of nanocrystals. While HRTEM gives information about the structure of single particles, selected area electron diffraction (SAED) and powder X‐ray diffraction (PXRD) allow the structural investigation of a statistically relevant ensemble of nanoparticles. Figure 1d,h are the corresponding indexed SAED patterns for Bi‐0% and Bi‐0.1%. In these patterns, all observed Debye–Scherrer rings were in accordance with the sole presence of In_2_O_3_ with a cubic structure. Identical SAED intensity profiles in Figure S1 (Supporting Information) indicated the same crystal structure and similar nanocrystal size for both pure and Bi substituted In_2_O_3−_ *_x_*(OH)*_y_*. This is consistent with the PXRD patterns showing no segregation of Bi‐containing crystal phases (Figure S2, Supporting Information). To further investigate the elemental distribution of In, O, and Bi, as well as the local configurations at the sub‐nanometer scale, the sample with the highest Bi substitution amount (Bi‐0.5%), was characterized using high angle annular dark field‐scanning transmission electron microscopy (HAADF‐STEM) combined with energy dispersive X‐ray spectroscopy (EDXS) and X‐ray absorption spectroscopy (XAS). The EDXS elemental maps of In‐L_α1_, Bi‐M_α_, and O‐K_α1_ (Figure 1j,l) show the homogenous distribution of In, Bi, and O atoms over the entire crystal network of the nanoparticle ensemble in the HAADF‐STEM image shown in Figure 1i. In agreement with the HRTEM, SAED, and PXRD experiments, no segregation of any Bi‐containing (crystalline or amorphous) phase was observed on the EDXS elemental map of Bi distribution. HAADF‐STEM image and EDXS elemental maps of a region of the sample with higher magnification (Figure S3, Supporting Information), wherein individual nanoparticles can be observed, further support that Bi is finely distributed over each nanoparticle and no Bi segregation is observed. From the integrated EDXS spectrum of the nanoparticle ensemble and substrate (Figure S4c, Supporting Information, red frame), a total chemical composition of Bi_0.3 ± 0.1_In_28.2 ± 1.5_O_71.5 ± 2.1_ is calculated. After substrate correction (Figure S4c, Supporting Information, blue frame), a real composition of Bi_0.4 ± 0.2_In_37.5 ± 2.0_O_62.1 ± 3.0_ is determined, which corresponds well to the formation of In_2_O_3_ nanoparticles (i.e., In_40_O_60_). The Bi L_3_‐edge and In K‐edge X‐ray absorption near‐edge (XANES) spectra (**Figure** **2**a,b) reveal that the Bi‐0.5% spectrum is similar to those of the corresponding Bi_2_O_3_ and In_2_O_3−_ *_x_*(OH)*_y_* (Bi‐0%) references, with Bi and In both being in the 3+ oxidation state. Examination of the Fourier‐transformed Bi L_3_‐edge extended X‐ray absorption fine structure (EXAFS) spectra from the Bi_2_O_3_ reference sample (Figure 2c) reveals a Bi—Bi interaction peak near 3.5 Å; this peak represents the shell of second‐nearest neighbors surrounding Bi atoms in the material, and does not line up with the Bi—M (M = Bi or In) peaks in the Bi‐0.5% sample. This difference indicates that the Bi atoms in Bi‐0.5% exist in a coordination environment different from that of the Bi_2_O_3_ reference material. Noticeably, direct comparisons between the Bi L_3_‐edge and In K‐edge Fourier‐transformed EXAFS spectra of Bi‐0.5% (Figure S5, Supporting Information), and In K‐edge Fourier‐transformed EXAFS spectrum of Bi‐0% (Figure 2d) reveal that the indium and bismuth atoms in Bi‐0.5% exist in a coordination environment similar to that of the indium atoms in Bi‐0%. Structural parameters of Bi‐0%, Bi‐0.5%, and Bi_2_O_3_ reference samples were quantitatively determined through fitting of EXAFS spectra (Figure S6, Supporting Information) as summarized in Table S1 (Supporting Information). In theory, In_2_O_3_ has the bixbyite crystal structure, with all indium cations surrounded by six oxygen atoms (CN_In—O_ = 6) and two distinct sets of adjacent polyhedral In—In neighbors (CN_In−In_ = 6, at a distance of ≈3.34 Å, and CN_In—In*_ = 6, at a distance of ≈3.83 Å).^27^ In the present study, the prepared Bi‐0% sample has longer In—In distances (3.36 and 3.84 Å) and lower coordination numbers (5.1 and 3.1), suggesting structural distortion caused by defects, which is consistent with the observed presence of surface hydroxide and oxygen vacancy.^28^ The spectra of the Bi‐0.5% sample is well fitted using a single Bi‐In scattering path to encompass both Bi—In and Bi—Bi scattering paths. The Bi—In and Bi—O distances and coordination numbers in Bi‐0.5% are close to those of In—In and In—O in Bi‐0%, providing further evidence that bismuth atoms have been successfully introduced into the In_2_O_3−_ *_x_*(OH)*_y_* network as individual, isolated atoms via isomorphous substitution for indium.^29^ The Bi—In and Bi—O distances are slightly longer than those of In—In and In—O, which is probably due to the larger ionic radius of Bi^3+^ (0.96 Å) relative to In^3+^ (0.81 Å).^30^ It is observed that the coordination number of In—O in Bi‐0.5% (CN = 5.6) is smaller than that in Bi‐0%, indicating that the introduction of Bi^3+^ into the lattice also affects the coordination environment of nearby In^3+^ atoms.

**Figure 3:**
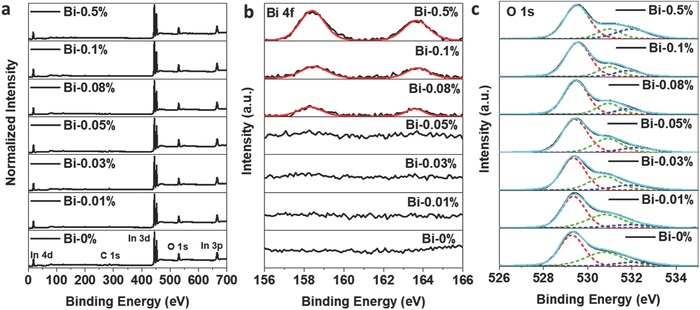
Comparison of XPS spectra from Bi*_z_*In_2−_ *_z_*O_3−_ *_x_*(OH)*_y_* samples. a) Normalized survey spectra for all samples. b) High‐resolution XPS spectra of the Bi 4f peak. c) Normalized O1s peaks with corresponding peak deconvolutions.

The surface properties of the prepared Bi*_z_*In_2−_ *_z_*O_3−_ *_x_*(OH)_y_ nanocrystals were then characterized via X‐ray photoelectron spectroscopy (XPS) analysis. No N1s peak can be observed in the XPS (Figure S7, Supporting Information), thus confirming the absence of nitrogen surface species and complete removal of nitrate ions during washing. The full XPS spectra of all samples are shown in **Figure** **3**a, while Figure 3b shows the Bi 4f core level XPS spectra. For Bi‐0.01%, Bi‐0.03%, and Bi‐0.05% samples, no peak from Bi can be observed due to the low substitution level, with the Bi concentration falling below the detection limit. From Bi‐0.08% to Bi‐0.5%, peaks begin to appear in the binding energy range of 156–166 eV and the peak intensity increases with the doping level; this is consistent with the inductively coupled plasma optical emission spectroscopy (ICP‐OES) result (Table S2, Supporting Information) showing that the Bi to In molar ratio increases in these samples. For the Bi‐0.5% sample, the atomic weight ratio of bismuth to indium measured by ICP‐OES (0.684%) is within the range calculated from the integrated EDX spectra (Bi_0.4 ± 0.2_In_37.5 ± 2.0_O_62.1 ± 3.0_). The relatively larger bismuth substitution value from ICP‐OES, relative to the nominal amount used in the synthesis, is probably due to the fact that Bi^3+^ hydrolyzes more easily than In^3+^, which results in less‐complete incorporation of In^3+^.^31^ In agreement with the previous XANES results, the occurrence of XPS peaks of Bi4f_7/2_ at 158.5 eV and Bi 4f_5/2_ at 163.5 eV demonstrated that the bismuth valence is +3 in these Bi*_z_*In_2−_ *_z_*O_3−_ *_x_*(OH)*_y_* samples.^32^ The O1s core level spectra of In_2_O_3−_ *_x_*(OH)*_y_* and Bi*_z_*In_2−_ *_z_*O_3−_ *_x_*(OH)*_y_* are summarized in Figure S8a (Supporting Information). Similar to our previously reported In_2_O_3−_ *_x_*(OH)*_y_* nanocrystals, a shoulder peak besides the main oxygen peak at 529.5 eV appears in the O1s core level spectra for all samples, corresponding to the surface defects that function as active sites for gas‐phase CO_2_ reduction.[[qv: 28b]] Further deconvolution of the O1s peak (Figure 3c) gives three distinct peaks, which can be assigned to the main oxygen peak (529.5 eV), oxygen vacancies (531.0 eV), and hydroxides (532.1 eV).^33^ With increasing Bi substitution level, the shoulder peak corresponding to surface defects from both vacancies and surface hydroxides decreases; however, there are exceptions to this trend, as Bi‐0.5% has slightly increased shoulder peak intensity relative to Bi‐0.1%, and Bi‐0.01% has the same shoulder peak intensity as Bi‐0% (Table S2, Supporting Information).

### 2.2 CO_2_ Reduction Activity

**Figure 4:**
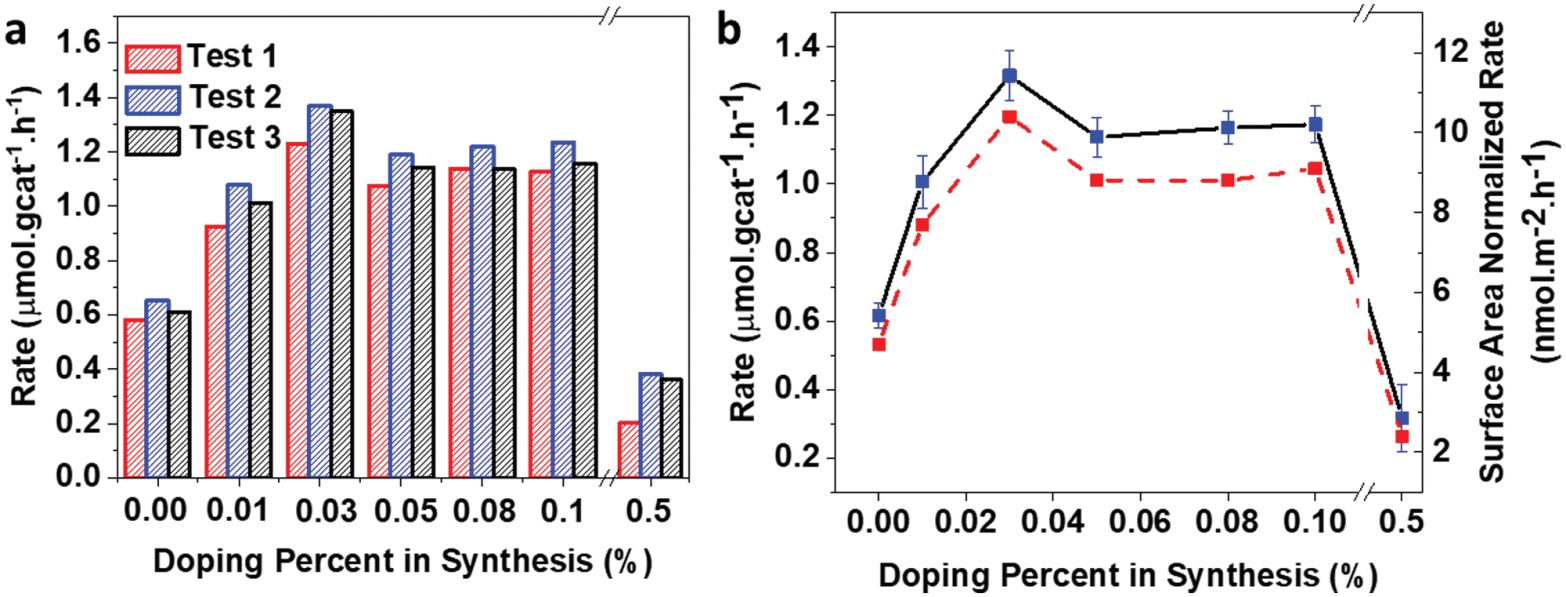
a) CO production rate from photocatalytic CO_2_ reduction on Bi‐0% to Bi‐0.5% samples. b) Plot of average CO production rate (black) and surface‐normalized CO production rate (red) as a function of the Bi doping level during synthesis.

To demonstrate the potential of Bi*_z_*In_2−_ *_z_*O_3−_ *_x_*(OH)*_y_* in enhancing the photocatalytic reduction of CO_2_, the light‐assisted activity of the above Bi‐0% to Bi‐0.5% samples for the RWGS reaction was investigated in a batch reactor filled with CO_2_ and H_2_, under 1 sun intensity illumination and at 150 °C. Isotope tracing experiments using ^13^CO_2_ with coupled gas chromatography‐mass spectroscopy (GC‐MS) analysis confirmed that the observed CO product originated from the ^13^CO_2_ feedstock, rather than from adventitious carbon sources (Figure S9, Supporting Information). All samples maintained good stability, even after three consecutive 12 h runs (**Figure** **4**a). Comparing the average CO production rates (Figure 4b), all Bi‐doped samples up to a nominal substitution concentration of 0.1% showed an increase in activity over the unsubstituted sample, with Bi‐0.03% exhibiting the highest activity (1.32 µmol g_cat_^−1^ h^−1^). Sample Bi‐0.5%, however, showed a markedly decreased CO production rate (Table S2, Supporting Information). The presence of fewer hydroxide groups and oxygen vacancy surface defects should have resulted in lower photocatalytic activity, due to a reduced number of active sites.^26^, ^33^ However, as shown in the XPS and CO production rate results, the CO production rate increases in accordance with a volcano‐shaped trend despite the decreasing number of defects between Bi‐0% and Bi‐0.1% (Figure 4b). Furthermore, Bi‐0.5% has only a slightly higher number of defects than Bi‐0.1%, but its CO production rate is even lower than that of In_2_O_3−_ *_x_*(OH)*_y_* (Bi‐0%). This suggests that the origin of the increase in CO_2_ reduction rate subsequent to Bi doping cannot be fully explained by defect concentration. Our previous study has indicated that the RWGS reaction on In_2_O_3−_ *_x_*OH*_y_* is a photochemical process.^26^ To check whether the CO production with Bi^3+^‐substituted In_2_O_3−_ *_x_*OH*_y_* is driven by photochemical or photothermal catalysis, a control experiment on the Bi‐0.1% sample was carried out in a flow reactor at three different temperatures: 150, 170, and 190 °C. The results in Figure S10a (Supporting Information) show that the CO production rates are all much higher with light irradiation than without light irradiation at the same temperature. Specifically, at 150 °C without light irradiation, no CO formation was observed for Bi‐0.1%. In order to contrast the activity with respect to light, an apparent activation energy was calculated. Assuming that the apparent reaction rate constant, *k*, is proportional to the CO production rate, a pseudo‐Arrhenius plot was prepared to estimate the apparent activation energy of both the thermal and photochemical reactions, as shown in Figure S10b (Supporting Information). The apparent activation energy of the photochemical RWGS reaction was estimated to be 120 kJ mol^−1^, whereas a value of 184 kJ mol^−1^ was estimated for the thermochemical reaction. These differences between light and dark activation energies and CO production rates indicate that the RWGS reaction on Bi^3+^‐substituted In_2_O_3−_ *_x_*OH*_y_* is likely a photochemical process as well. Thus, the optoelectronic property of the catalyst plays an important role in this catalytic process.

### 2.3 Optoelectronic Characterization

**Figure 5:**
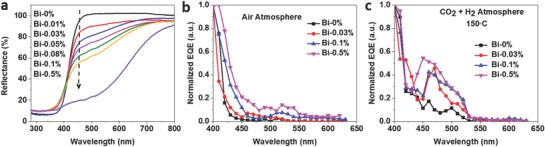
a) Diffuse reflectance spectra of the as‐prepared nanocrystal films used in photocatalysis experiments. EQE spectra of Bi‐0%, Bi‐0.03%, Bi‐0.1%, and Bi‐0.5% samples b) in air at room temperature and c) in a CO_2_ and H_2_ gas mixture at 150 °C.

Composite oxides containing Bi^3+^ usually exhibit a strong response to visible light due to the hybridization between Bi 6s and O 2p orbitals, resulting in an upward shift of the valence band and a reduced bandgap.^34^, ^35^ Bi^3+^‐doped NaTaO_3_ and TiO_2_ have been reported to demonstrate improved organic molecule photodegradation activity due to increased visible light absorption.^36^, ^37^ The Bi*_z_*In_2−_ *_z_*O_3−_ *_x_*(OH)*_y_* nanocrystals prepared herein showed similar Bi 6s orbital effects, with the color becoming increasingly dark (Figure S11, Supporting Information). Diffuse reflectance spectroscopy (**Figure** **5**a) showed that the calculated bandgap (Table S2, Supporting Information) also decreased with larger amounts of Bi. To further compare their optoelectronic properties, wavelength‐dependent photoconductivities were assessed using an external quantum efficiency (EQE) measurement system. The EQE spectrum represents the charge carrier collection, or harvesting, efficiency of the material when subjected to photon radiation. Electron carriers introduced into the conduction band under the appropriate wavelengths of incident light diffuse through the nanocrystal system and are harvested through electrode pads. Whether there are free carriers that can be excited by sub‐bandgap energies depends on the energy distribution of trap states, and the photocurrent so generated depends on their density and detrapping activation energy values.^38^, ^39^, ^40^ From the EQE spectrum in air (Figure 5b), a nonzero photocurrent was detected past the main photocurrent edge for In_2_O_3−_ *_x_*(OH)*_y_*, which indicated that shallow to deep trap states were present up to 550 nm (2.25 eV).^41^, ^42^ With increasing Bi substitution levels, the main photocurrent edge increased from 430 to 470 nm, while the photocurrent density in the sub‐bandgap energy range at above ≈500 nm is the highest for Bi‐0.5%. This indicates that the Bi‐0.5% sample has deep states that likely extend past 640 nm (1.94 eV), consistent with the UV–vis diffuse reflectance result. To mimic the catalytic reaction process and compare optoelectronic properties under the catalytic reaction conditions, wavelength‐dependent photoconductivities were measured under a mixture of CO_2_ and H_2_ gases (1:1 molar ratio) at 150 °C. Figure 5c shows that Bi‐0.5% still had the highest photoresponse in the visible range, despite having the lowest photocatalytic performance.^43^ Meanwhile, Bi‐0.03% had the weakest photoresponse among all the Bi‐substituted samples studied herein, but proved to be the most active in comparison with the Bi‐0.1% and Bi‐0.5% samples. Such a lack of correlation between the exhibited photocatalytic activity and the trends in optoelectronic properties, and concentration of oxygen surface defects, suggests that the active surface sites of the photocatalyst require an optimal interaction with the reactants to be able to efficiently utilize the photoexcited electrons and holes for catalytic reactions; it may be necessary to consider the nature and reactivity of these defects, and not just their quantity.^44^ Our recent DFT studies into the effect of light on In_2_O_3−_ *_x_*(OH)*_y_* in the RWGS reaction showed that, with light irradiation, the Lewis acidity and Lewis basicity of surface FLPs increased in the excited state. Furthermore, the resulting proton and hydride formed by the heterolytic splitting of H_2_ on the In—OH···In surface FLPs became more protonic and hydridic, respectively, leading to enhanced photocatalytic activity relative to their activity in the dark.^25^, ^42^ As CO_2_ only reacts on hydrogenated In_2_O_3−_ *_x_*(OH)*_y_* surfaces bearing both a hydride and a proton, DFT calculations were performed to focus on the H_2_ dissociation step, in order to understand the effect of Bi^3+^ substitution on the surface FLPs.^26^, ^45^ Bader charge analysis was utilized to ascertain the relative charges associated with individual atoms on the surface near the FLP site before and after H_2_ dissociation.

### 2.4 DFT Calculations

**Figure 6:**
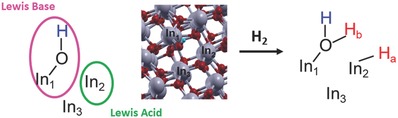
In_2_O_3−_ *_x_*(OH)*_y_* surface showing a surface FLP site, wherein In, O, and H atoms are represented by grey, red, and blue spheres, respectively.

**Figure 7:**
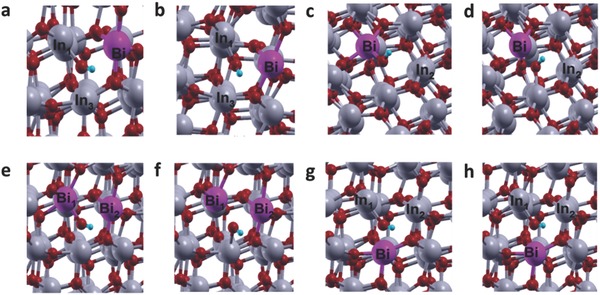
a) Initial and b) final configurations of the surface with Bi substitution at the acid site of the surface FLP. c) Initial and d) final configurations of the surface with Bi substitution at the base site of the surface FLP, e) initial and f) final configurations of the surface with Bi substitution at both base and acid sites of the surface FLP, and g) initial and h) final configurations of the surface with Bi substitution at the neighboring site of the surface FLP. In, O, H, and Bi atoms are represented by gray, red, blue, and purple spheres, respectively.

Four configurations of Bi substitution at the In_2_O_3−_ *_x_*(OH)*_y_* surface were considered: I – Bi substitution at the acid site of the surface FLP; II – Bi substitution at the base site of the surface FLP; III – Bi substitution at both base and acid sites of the surface FLP; and IV – Bi substitution at the neighboring In site of the surface FLP. In_1_, In_2_, and In_3_ represent three different indium atoms that can be substituted by bismuth to alter the In—OH···In surface FLP site (**Figure** **6**). In configuration I, the In_2_ site of the In_2_O_3−_ *_x_*(OH)*_y_* surface is substituted by Bi, leading to the initial configuration having In_1_—OH as the base site and Bi as the acid site (**Figure** **7**a). Upon relaxation, it was found that OH binds strongly to the In_1_ atom (bond length = 2.17 Å) just as in the case of unsubstituted surface (bond length = 2.19 Å). The relaxed structure for the In—OH···Bi surface is shown in Figure 7b. Bader charge analysis revealed that the Lewis acidic Bi and Lewis basic O of the OH sites at the surface possessed charges of +2.65e and −1.38e, respectively. On comparison with the charges of the Lewis acidic In_2_ (+1.55e) and Lewis basic O (−1.50e) of the OH sites of the unsubstituted FLP, one can see that Bi substitution at the acid site greatly increased the acidity, but simultaneously decreased the basicity of the surface FLP. In configuration II, In_1_ of the surface FLP is substituted with Bi, leading to the initial configuration having Bi—OH as the base site and In_2_ as the acid site (Figure 7c). Upon relaxation, it was found that, instead of binding to surface Bi atom, OH preferred to bond to the neighboring In atom (In_3_) (Figure 7d). A similar phenomenon was observed for configuration III, wherein both the base site, In_1_, and the acid site, In_2_, of the surface FLP were substituted with Bi, leading to the initial configuration Bi_1_—OH···Bi_2_ (Figure 7e). Upon relaxation, the Bi_1_—OH bond breaks and the OH binds to indium at In_3_, resulting in a Bi_1_···Bi_2_···In_3_—OH surface (Figure 7f). In configuration IV, Bi substitutes the In_3_ atom, leading to the initial configuration having In_1_—OH as the base and In_2_ as the acid site, with Bi substituted at the neighboring site of this FLP (Bi···In_1_—OH···In_2_) (Figure 7g). Interestingly, when this system was relaxed (Figure 7h), the bond length of In_1_—OH (2.175 Å) remained similar to the case of the unsubstituted surface (Figure S11, Supporting Information). Bader charge analysis revealed that, under these conditions, the basicity of the base site increased (the charge on O of OH was −1.69e for the substituted system vs −1.50e for the unsubstituted system), likely as a result of the Bi 6s^2^ lone pair at the neighboring site transferring electrons to the OH group.^46^ The acidity of In_2_ maintained the same charge (+1.55e). In this case, both In_2_ and Bi (charge = +2.52e) can act as acid sites. The higher charge observed in Bi is probably due to the lower molar ionization energy, which allows bismuth to lose electrons and obtain high oxidation charge more easily than indium.^30^ Upon comparing the Bader charges of these Lewis acidic In (+1.55e) and Lewis basic O (−1.50e) of the OH sites to those of the unsubstituted FLP site (Figure S10, Supporting Information), one can see that Bi substituted at the neighboring site may lead to an overall increase in activity of the surface FLPs relative to the unsubstituted system, due to a simultaneous increase in both the basicity and acidity of the FLP site. Due to the instability of the Bi—OH structure in configurations II and III, only configurations I and IV are considered for further catalysis steps.

**Figure 8:**
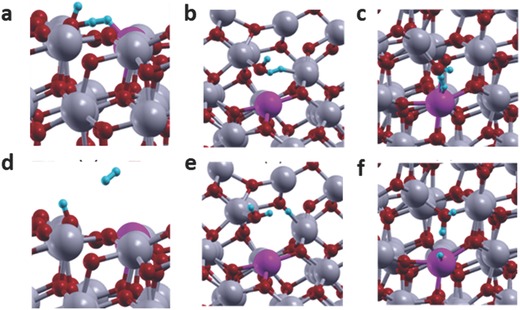
a) Initial and d) final configurations of H_2_ adsorption at the In—OH···Bi FLP site with enhanced acidity due to Bi‐substitution at the acid site. b) Initial and e) final configurations of H_2_ adsorption on In_1_—OH···In_2_ site having enhanced basicity, but the same acidity, as the unsubstituted system due to Bi‐substitution at the In_3_ neighboring site. c) Initial and f) final configurations of H_2_ adsorption on In_1_—OH and Bi site with both enhanced acidity as basicity of the surface FLP due to Bi substitution at the In_3_ neighboring site.

To examine the interaction of gaseous H_2_ with the Bi substituted surfaces having altered acidity and basicity, H_2_ adsorption on the following surfaces was further analyzed:

(a) In_1_—OH···Bi_2_ surface having enhanced Lewis acidity of the surface FLP (**Figure** **8**a)
(b) Bi_3_···In_1_—OH···In_2_ surface having enhanced Lewis basicity but the same Lewis acidity of the surface FLP (as in the case of the unsubstituted system, Figure 8b)
(c) Bi_3_···In_1_—OH···In_2_ surface having both enhanced Lewis acidity and enhanced Lewis basicity of the surface FLP (Figure 8c).

The DFT analyses show that when H_2_ was placed on the In_1_—OH···Bi_2_ site (case a), it failed to dissociate and remained intact. This is contrary to the H_2_ interaction on the unsubstituted surface, which results in heterolytic H_2_ splitting at the In_1_—OH···In_2_ FLP site. This result clearly indicated that, due to the decreased Lewis basicity of In—OH, Bi substitution at the acid site could not enhance the activity of surface FLPs, despite the greatly increased Lewis acidity.^47^ When H_2_ was placed in the vicinity of either the In_1_—OH···In_2_ (case b) or In_1_—OH···Bi_3_ (case c) FLP sites formed by Bi substitution at the neighboring site, the enhanced acidity and basicity of these FLP sites generated a stronger electric field that polarized the H_2_ molecule as it approached the surface and led to the heterolytic dissociative adsorption of H_2_ forming oppositely charged hydride and proton.^47^ The bond lengths and Bader charges for these two cases (b and c) and the unsubstituted surface are provided in **Table** **1**. It is also interesting to observe that the resulting hydride is more hydridic in both case b and case c. The extra negative charge on the hydride facilitates the charge transfer from the catalyst surface to CO_2_, causing the C—O bond to lengthen and break.^42^, ^45^, ^48^

**Table 1:** Bader charge and structural analyses following H_2_ dissociation on the unsubstituted In_2_O_3−_ *_x_*(OH)*_y_* surface, with the substituted surface having Bi at the neighboring site of the surface FLP

Surfaces	Bader charge (e) after H_2_ splitting	Bond length [Å]
H_2_ in the vicinity of In_1_—OH···In_2_ site on unsubstituted surface	H_a_, H_b_, O, H, In_1_, In_2_, In_3_−0.36, +0.74, −1.56, +0.76, +1.59, +1.65, +1.61	H_2_O—In_1_: 2.42In_2_—H: 1.80
		
H_2_ in the vicinity of In_1_—OH···In_2_ site on the surface having In_3_ substituted by Bi (case b)	H_a_, H_b_, O, H, In_1_, In_2_, Bi−0.39, +0.63, −1.44, +0.75, +1.67, +1.60, +2.73	H_2_O—In_1_: 2.41In_2_—H: 1.82
		
H_2_ in the vicinity of In_1_—OH···Bi site on the surface having In_3_ substituted by Bi (case c)	H_a_, H_b_, O, H, In_1_, In_2_, Bi−1.15, +0.63, −1.35, +0.64, +1.76, +1.57, +2.88	H_2_O—In_1_: 2.28Bi_3_—H: 2.02
		

## 3 Conclusion

In pursuing heterogeneous catalysts capable of facilitating the efficient conversion of gaseous CO_2_ to useful chemicals and fuels, it has been demonstrated that the nature of surface FLP sites can serve as an effective descriptor for identifying the best catalyst in an isostructural series of materials. This strategy is exemplified herein by the archetypical material Bi*_z_*In_2−_ *_z_*O_3−_ *_x_*(OH)*_y_*, which catalyzes the photochemical RWGS (CO_2_ + H_2_ → CO + H_2_O). By varying the concentration of Bi^3+^ in the lattice of nanocrystalline In_2_O_3−_ *_x_*(OH)*_y_*, a maximum in the “volcano” plot for the conversion of CO_2_‐to‐CO was found to occur at *z* = 0.0003. Density functional theory calculations suggested that the uphill portion of the “volcano” curve can be traced to the increase in both Lewis acidity and Lewis basicity of the FLP when Bi substitutes at the neighboring In_3_ site. Initially, this serves to improve the heterolytic splitting of reactant H_2_, whereupon the rate of the RWGS reaction increases. When the Bi‐substitution was further increased, acidic In_2_ atoms could also have been substituted by Bi, leading to a decrease in reaction rate due to a reduction in the FLP site strength, thereby giving rise to the downhill portion of the “volcano” plot. This is consistent with the experimental observation that the CO_2_ reduction rate decreased rapidly at higher Bi concentrations (i.e., 0.5%), even though the number of oxygen vacancy and surface hydroxide defects was high and the optoelectronic properties were enhanced. Based on the results of this study, it is anticipated that the strength of surface FLP site, which can be effectively tuned through isomorphous substitution, will become an increasingly valuable parameter in the search for CO_2_ reduction materials exhibiting superior catalytic performance.

## 4 Experimental Section

*Preparation of In_2_O_3−x_(OH)_y_*: In_2_O_3−_ *_x_*(OH)*_y_* nanocrystals, denoted as “Bi‐0%,” were prepared via dehydroxylation of In(OH)_3_ in air at 250 °C for 6 h. In a typical synthesis of In(OH)_3_, indium(III) nitrate hydrate (1.8 mmol, 0.54 g) was dissolved in ethanol (6 mL), to which was then added an ammonia solution prepared by mixing ammonium hydroxide (27%, 2.5 mL) with ethanol (7.5 mL) and water (2 mL). The resulting suspension of In(OH)_3_ was kept in a preheated oil bath at 80 °C for 10 min and then cooled to room temperature before the precipitate was collected via centrifugation and washed with deionized water. The resulting solid was dried at 60 °C in a vacuum oven.

*Preparation of Bi‐Doped In_2_O_3−x_(OH)_y_*: A series of Bi^3+^‐substituted In_2_O_3−_ *_x_*OH*_y_* nanocrystals (Bi*_z_*In_2−_ *_z_*O_3−_ *_x_*(OH)*_y_*, where *z* = 0.0003, 0.0005, 0.0008, 0.001, or 0.005, representing the nominal molar ratio of bismuth to indium in the synthesis) were prepared via dehydroxylation of Bi^3+^‐substituted In(OH)_3_ in air at 250 °C for 6 h. Samples are denoted as Bi‐0.03%, Bi‐0.05%, Bi‐0.08%, Bi‐0.1%, and Bi‐0.5%, respectively. Bi^3+^‐substituted In(OH)_3_ was prepared using the same method employed for In(OH)_3_, except that various amounts of the bismuth solution were added to the indium solution prior to addition of the ammonia solution. The bismuth solution (9 mmol L^−1^) was prepared by dissolving bismuth(III) nitrate pentahydrate (0.45 mmol, 0.22 g) in diluted nitric acid solution (50 mL).

*Characterization and Equipment*: PXRD was carried out on a Bruker D2‐Phaser X‐ray diffractometer using Cu Kα radiation, operating at 30 kV, in the 2θ range of 20°–70°, with a 2θ step size of 0.02° and a step time of 1 s. HRTEM images and SAED patterns were acquired on an aberration‐corrected FEI Titan^3^ 80‐300 microscope operated at a 300 kV accelerating voltage. HRTEM images were also evaluated by calculating their 2D Fourier transform (FT), yielding information regarding the crystal structure (i.e., lattice parameters and crystal symmetry) of single nanoparticles. The analysis was performed by comparing the experimental FT and calculated diffraction patterns for specific Miller indices, where the latter were obtained using the JEMS software.^49^ ZB indicates the zero‐order beam. HAADF‐STEM combined with EDXS was carried out on a FEI Osiris ChemiSTEM microscope operated at a 200 kV electron acceleration voltage, also equipped with a Bruker Quantax system (XFlash detector) for EDXS. EDXS elemental maps of In (In‐L_α1_), O (O‐K_α1_), and Bi (Bi‐M_α_) were recorded and used to investigate elemental distributions within the nanoparticles. Integrated EDXS spectra of mapped areas, including inside an ensemble of nanoparticles and on the backgroud subtrate, were quantified using ESPRIT software (version 1.9) from Bruker. XANES and EXAFS measurements were performed at the Advanced Photon Source of Argonne National Laboratory, Lemont, IL. Spectra were acquired at the Bi L_3_‐ and In K‐edges using the Sector 20‐BM beamline, with metal foil references used for energy calibration. XANES spectra were background‐subtracted and normalized to facilitate analysis, and EXAFS data fitting was performed using WinXAS software in conjunction with scattering functions generated by FEFF8 and crystal structures obtained from the Crystallographic Open Database.^50^ With the exception of the Bi_2_O_3_ reference, three principal scattering paths were included in the fits of both the Bi L_3_‐ and In K‐edge spectra: Bi/In—O, Bi/In—In, and Bi/In—In. These paths taken from an In_2_O_3−_ *_x_*(OH)*_y_* structural model were found to account for the majority of the observed EXAFS signal, and resulted in good fits to the data. Two scattering paths obtained from an α‐Bi_2_O_3_ structural model were used for fitting the Bi_2_O_3_ spectrum: Bi—O and Bi—Bi. Specific surface areas of all samples were determined from nitrogen Brunauer–Emmet–Teller (BET) adsorption isotherms on a Quantachrome Autosorb‐1‐C. The CO_2_‐capture capacity of each sample was measured via thermogravimetric analysis (TGA) using a TA Instruments – The Discovery TGA system. All UV–vis–NIR diffuse reflectance spectra were recorded over the spectral range of 250–800 nm on a Lambda 1050 UV––vis–NIR spectrometer from Perkin Elmer, which was equipped with an integrating sphere of diameter 150 mm. The optical bandgap was determined by fitting the diffuse reflectance spectra with a modified Kubelka–Munk function. Wavelength‐dependent photoconductivities were measured on a home‐built EQE measurement setup comprising a Keithley 2440 source meter, Spectra Product CM110 monochromator, 200 W Hg/Xe lamp, Princeton Model 5210 lock‐in amplifier, and a beam splitter. A chopper was used to conduct a four‐point probe *I*–*V* measurement over an incident wavelength range of 650–420 nm. The conductance value at each wavelength point was taken under illumination and normalized over the incident power to determine the EQE. XPS was conducted on a Perkin Elmer Phi 5500 ESC spectrometer using an Al Kα X‐ray source operating at 15 kV and 27 A. The binding energy was corrected using the C1s line at 284.5 eV. ICP‐OES was carried out on a Thermo Scientific iCAP 7000 Series ICP Spectrometer.

*Photocatalytic Measurements*: Gas‐phase photocatalytic CO_2_ reduction measurements (for the RWGS reaction) were conducted in a custom‐built 1.5 mL stainless steel batch reactor with a fused silica view port sealed with Viton O‐rings. The reactor was evacuated using an Alcatel dry pump prior to being purged with the reactant H_2_ gas (99.9995%) at a flow rate of 15 mL min^−1^. After purging the reactor, it was filled with a 1:1 stoichiometric mixture of H_2_ (99.9995%) and CO_2_ (99.999%) until the total pressure reached 30 psi. The reactor was then irradiated with a 1000 W Hortilux Blue metal halide bulb for a period of 12 h at a temperature of 150 °C. Product gases were analyzed using flame ionization and thermal conductivity detectors installed in a SRI‐8610 gas chromatograph equipped with a 3 in. Mole Sieve 13a and 6 in. Haysep D column. Isotopically labeled tracing experiments were performed using ^13^CO_2_ (99.9 at%, Sigma‐Aldrich). Isotope distributions in the product gases were measured using an Agilent 7890A gas chromatograph‐mass spectrometer with a 60 m GS‐carbonplot column leading to the mass spectrometer.

Gas‐phase rate measurements (both thermo‐ and photocatalytic) were carried out in a fixed‐bed tubular reactor. The reactor housing was a borosilicate tube (3 mm outer diameter and 2.5 mm inner diameter). Within the reactor tube, 20 mg of catalyst was packed between two portions of quartz wool, which supported the packed catalyst bed in the center. External heating was provided by conduction via a heated copper block surrounding the tubular reactor on all but one side. An OMEGA CN616 6‐Zone temperature controller managed the temperature utilizing a thermocouple placed in close proximity to the catalyst bed. Each temperature set point was maintained for 4 h before increasing it to the next set point. During the reaction, H_2_ (Praxair 99.999%) and CO_2_ (Praxair 99.999%) were flowed in a 1:1 ratio at a total volumetric flow rate of 2 sccm. For photocatalytic rate measurements, the reactor was irradiated with a 300 W Newport Xe lamp. An 8610 SRI Gas Chromatograph (10 Mol Sieve 5a column and a 60 Haysep D column) was used to identify and quantify product gases.

*Computational Details*: Spin‐polarized DFT calculations were performed using the Perdew–Burke–Ernzerhof (PBE) exchange‐correlation functional within the generalized gradient approximation (GGA), as implemented in the Quantum Espresso software.^51^ Kinetic energy cutoffs of 50 and 200 Ry were used for the wavefunctions and charge density, respectively, and the self‐consistent field convergence criterion was set to 10^−6^ Ry. Each system was relaxed with a variable cell size using conjugate gradient minimization until the magnitude of the residual Hellman–Feynman force on each atom was less than 10^–3^ Ry Bohr^−1^. Based on previous experimental evidence, DFT computations were restricted to the (111) surface in the present study.^26^ Notably, this surface is also the most abundant crystal face from a thermodynamic equilibrium perspective.^52^ To model the cubic In_2_O_3−_ *_x_*(OH)*_y_* (111) surface, a four‐layer slab containing 160 atoms was used, in which a vacuum layer of ≈20 Å was applied to avoid interaction between periodic images. The modeled system was a continuous layer roughly 11.5 Å in thickness and represented a nanofilm, capturing the behavior of the non‐edge nanocrystal regions that form the majority of the surface area. In all calculations, the bottom two layers were frozen at their equilibrium bulk positions (after bulk relaxation), while the top two layers and the adsorbates were allowed to relax. Because of the relatively large size of the supercell, Brillouin zone integrations were performed using the gamma k‐point only. The side and the top views of the In_2_O_3−_ *_x_*(OH)*_y_* supercell are shown in Figure S10a,b (Supporting Information), respectively. To quantify the charge transfer, Bader charge analyses were performed for the Bi‐substituted and unsubstituted surfaces.^53^, ^54^

## Conflict of Interest

The authors declare no conflict of interest.

## Supporting information

Supplementary
