# Supplementary material for: Tailoring Surface Frustrated Lewis Pairs of In2O3− x(OH)y for Gas‐Phase Heterogeneous Photocatalytic Reduction of CO2 by Isomorphous Substitution of In3+ with Bi3+
Source: Adv Sci (Weinh). 2018 Mar 12;5(6):1700732. doi: 10.1002/advs.201700732 (PMC6009996; doi:10.1002/advs.201700732)
Supplement: Supplementary file 1 — Supplementary [file ADVS-5-1700732-s001.pdf]

Copyright WILEY-VCH Verlag GmbH & Co. KGaA, 69469 Weinheim, Germany, 2018.

ADVANCED SCIENCE
Open Access

Supporting Information

for *Adv. Sci.,* DOI: 10.1002/advs.201700732

Tailoring Surface Frustrated Lewis Pairs of $In_2O_{3-x}(OH)_y$ for Gas-Phase Heterogeneous Photocatalytic Reduction of CO_2 by Isomorphous Substitution of In^{3+} with Bi^{3+}

Yuchan Dong, Kulbir Kaur Ghuman, Radian Popescu, Paul N. Duchesne, Wenjie Zhou, Joel Y. Y. Loh, Abdinoor A. Jelle, Jia Jia, Di Wang, Xiaoke Mu, Christian Kübel, Lu Wang, Le He, Mireille Ghoussoub, Qiang Wang, Thomas E. Wood, Laura M. Reyes, Peng Zhang, Nazir P. Kherani, Chandra Veer Singh, and Geoffrey A. Ozin**

Copyright WILEY-VCH Verlag GmbH & Co. KGaA, 69469 Weinheim, Germany, 2016.

Supporting Information

Title: Tailoring Surface Frustrated Lewis Pairs of $In_2O_{3-x}(OH)_y$ for Gas-Phase Heterogeneous Photocatalytic Reduction of CO_2 by Isomorphous Substitution of In^{3+} with Bi^{3+}

Yuchan Dong,† Kulbir Kaur Ghuman,† Radian Popescu, Paul N. Duchesne, Wenjie Zhou, Joel Y. Y. Loh, Abdinoor A. Jelle, Jia Jia, Di Wang, Xiaoke Mu, Christian Kübel, Lu Wang, Le He, Mireille Ghoussoub, Qiang Wang, Thomas E. Wood, Laura M. Reyes, Peng Zhang, Nazir P. Kherani, Chandra Veer Singh, Geoffrey A. Ozin**

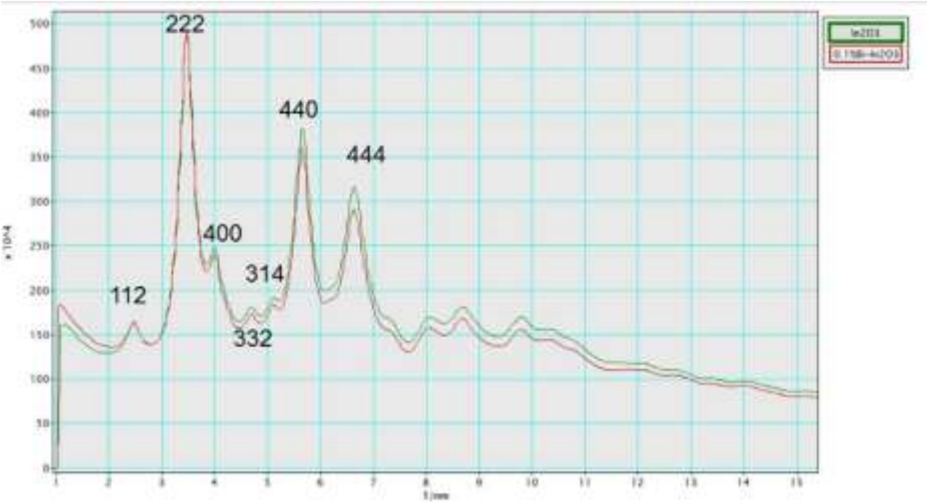

Figure S1. SAED intensity profiles for Bi-0% ($In_2O_{3-x}(OH)_y$) and Bi-0.1%, indicating the same crystal structure and similar particle size.

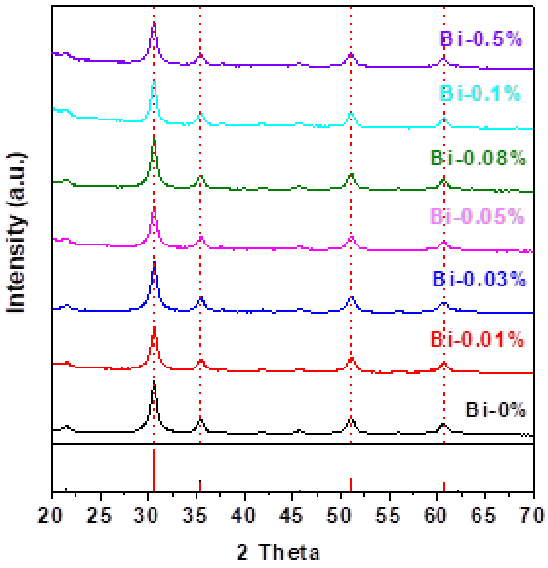

Figure S2. Comparison of PXRD patterns obtained from $In_2O_{3-x}(OH)_y$ and $Bi_zIn_{2-z}O_{3-x}(OH)_y$ with different Bi substitution levels.

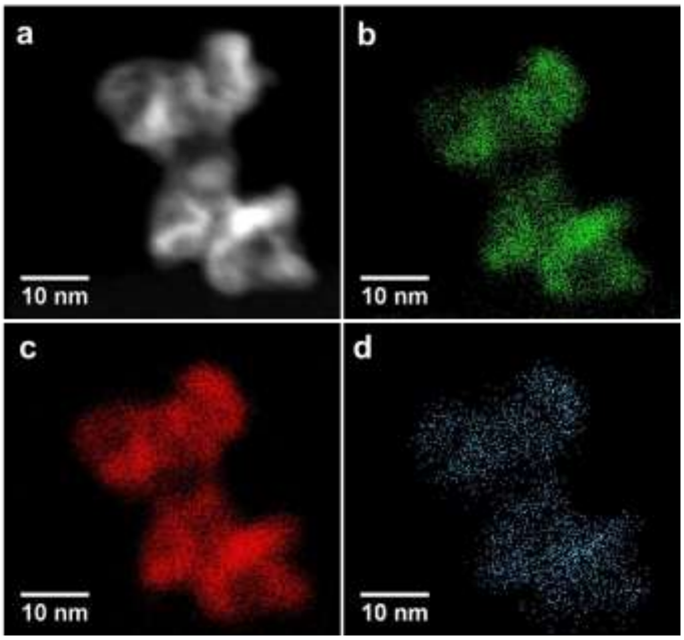

Figure S3. High-magnification HAADF-STEM image (a) and EDXS elemental maps of (b) O (O-$K_{\alpha1}$ green), (c) In (In-$L_{\alpha1}$ red) and (d) Bi (Bi-M_{α} blue) for nanoparticles belonging to the Bi-0.5% sample, wherein the homogeneous distribution of O, In and Bi elements within individual nanoparticles can be observed.

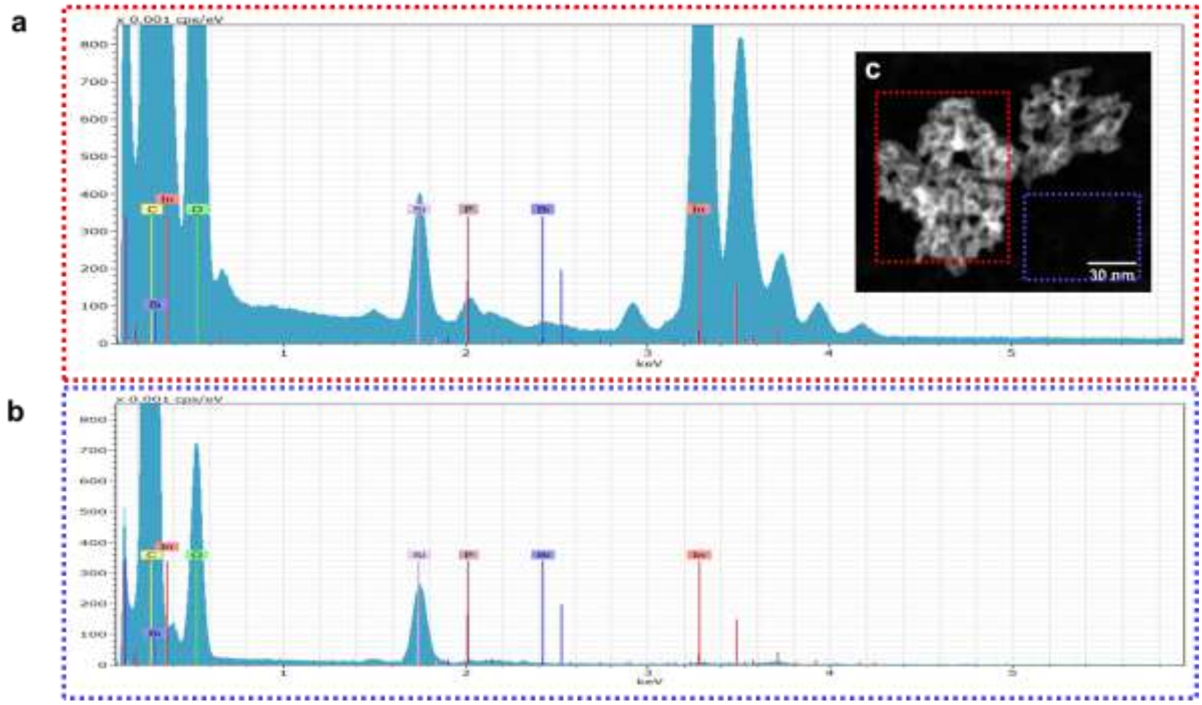

Figure S4. From the Bi-0.5% sample: (a) integrated EDXS spectra of the nanoparticle ensemble within the red frame of the HAADF-STEM image (c), and (b) integrated EDXS spectrum of the substrate within the blue frame of the HAADF-STEM image (c).

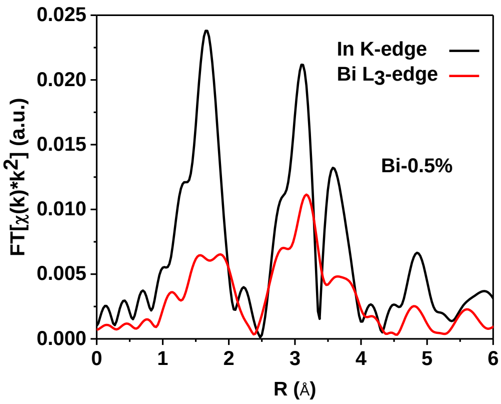

Figure S5. Fourier-transformed EXAFS spectra of Bi-0.5%.

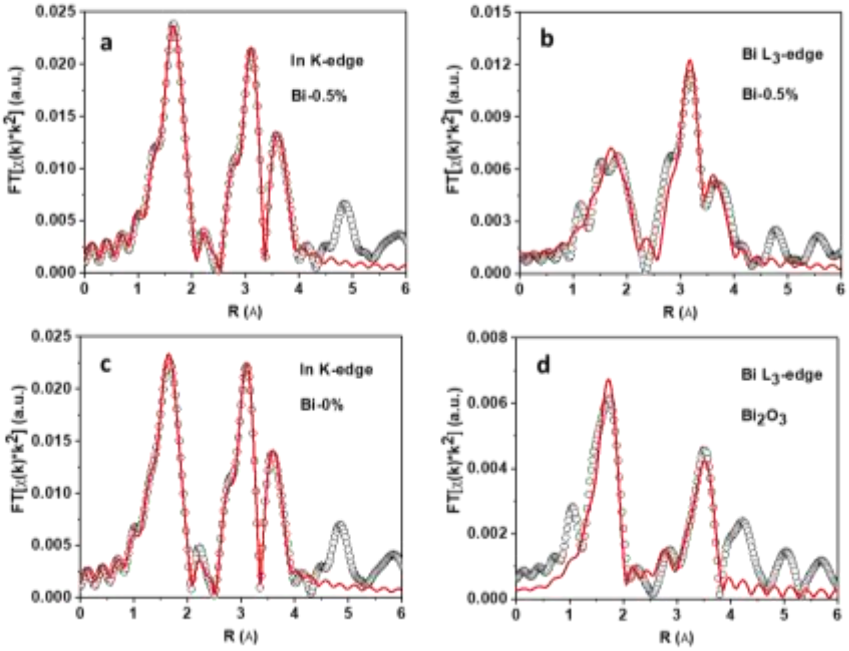

Figure S6. Experimental (open circles) and fitted (solid red line) Fourier-transformed EXAFS spectra used in determining the occupancy of the atomic positions in the vicinity of the In and Bi ions.

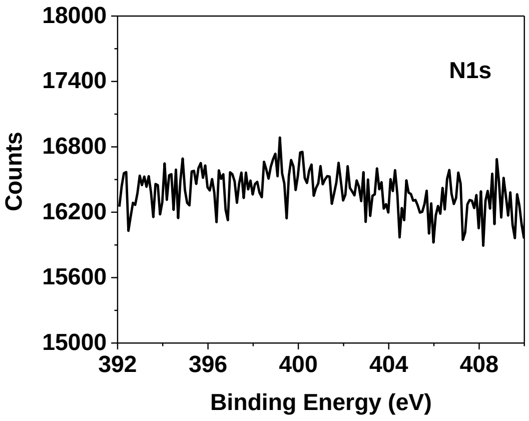

Figure S7. N1s peak for the Bi-0.1% sample

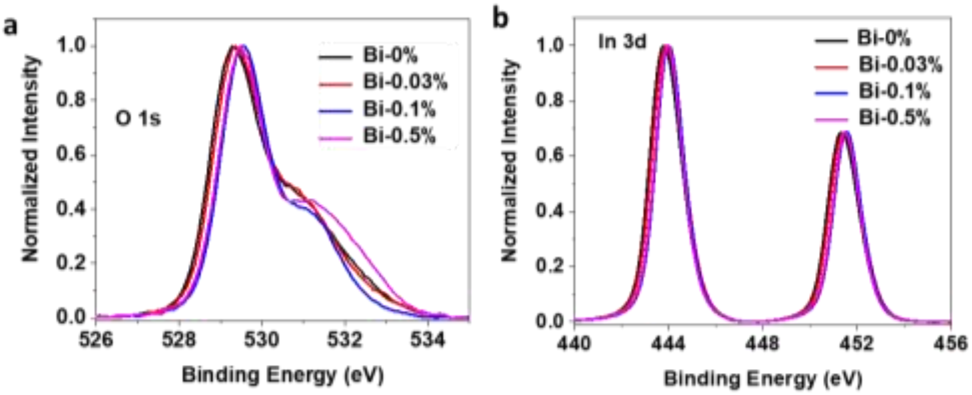

Figure S8. Comparison of XPS spectra for different $Bi_zIn_{2-z}O_{3-x}(OH)_y$ samples. (a) Normalized high resolution XPS spectra of the O1s and (b) In 3d peaks for Bi-0%, Bi-0.03%, Bi-0.1% and Bi-0.5%.

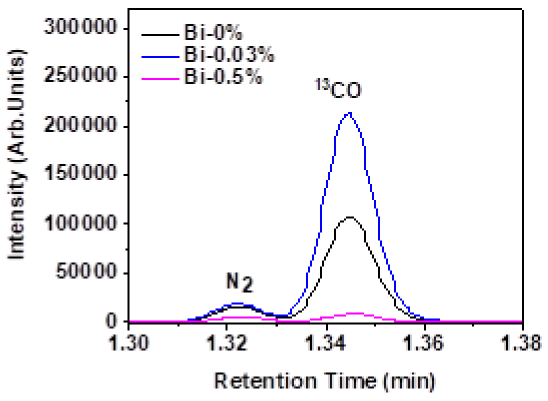

Figure S9. Mass spectrum of photo-thermally generated ^{13}CO from $^{13}CO_2$. The AMU 29 mass fragment peak at approximately 1.345 min corresponds to ^{13}CO.

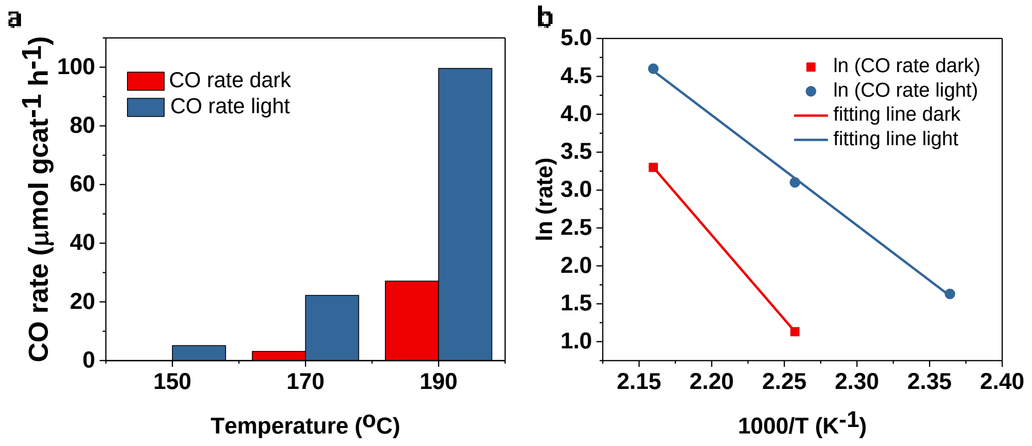

Figure S10. (a) Temperature dependence of CO production for the Bi-0.1% sample under flowing H_2 and CO_2. (b) Pseudo-Arrhenius plots drawn from temperature dependent measurements.

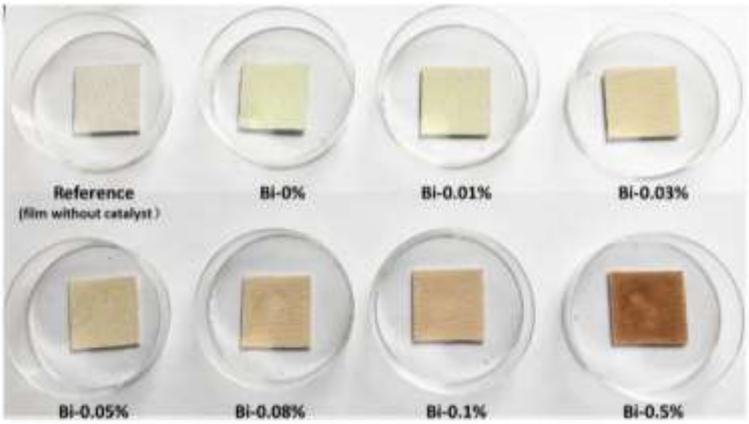

Figure S11. Photograph of each film sample used for UV-VIS-NIR diffuse reflectance spectroscopy and gas-phase photocatalysis measurements. The film samples were prepared by drop-casting aqueous dispersions of catalyst nanocrystals onto borosilicate glass microfiber filters. The reference sample is the glass microfiber filter.

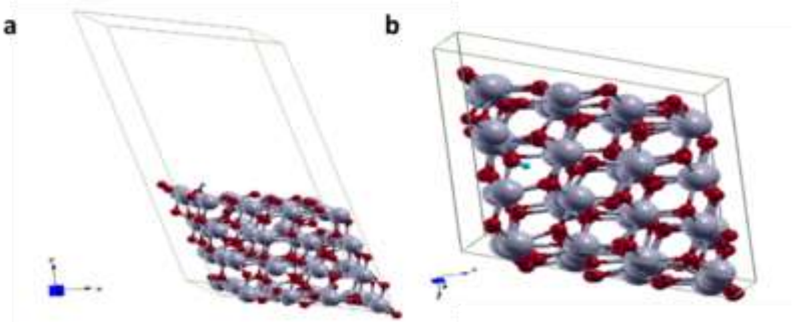

Figure S12. (a) Side and (b) top view of the $In_2O_{3-x}(OH)_y$ supercell used for the DFT calculation.

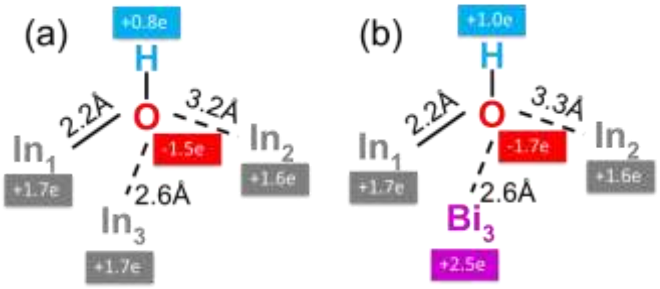

Figure S13. Schematic diagram showing bond lengths and charges for (a) unsubstituted, and (b) Bi-substituted $Bi\cdots In_1\text{-}OH\cdots In_2$ surfaces.

Table S1. Structural parameters and (in parentheses) estimated uncertainties from EXAFS of Bi-0% (*i.e.*, unsubstituted $In_2O_{3-x}(OH)_y$), Bi-0.5% and reference Bi_2O_3 samples.

**Sample**	**Edge**	**Path**	**CN**	**R (Å)**	**σ^2 (10^{-3} Å^2)**	**ΔE_0 (eV)**
Bi-0%	In K	In–O	6.0(2)	2.171(4)	5.6(6)	–0.1(3)
		In–In	5.1(5)	3.361(2)	3.1(4)	
		In–In	3.1(6)	3.845(3)	1.7(9)	
Bi_2O_3	Bi L_3	Bi–O	5.5*	2.17(1)	3(2)	–4(1)
		Bi–Bi	8(3)	3.59(1)	4(2)	
Bi-0.5%	In K	In–O	5.6(2)	2.171(3)	4.9(5)	0.7(3)
		In–In	5.4(4)	3.363(2)	3.6(4)	
		In–In	2.9(5)	3.848(3)	1.4(8)	
	Bi L_3	Bi–O	6(2)	2.28(2)	14(6)	–5(2)
		Bi–In	5(1)	3.44(1)	4(1)	
		Bi–In	2(1)	3.87(2)	1**	

* *This parameter value was held fixed during fitting and has no associated uncertainty.*

** *Due to a high degree of correlation between parameters, uncertainty could not be determined for this value.*

Table S2. Summary of properties of $In_2O_{3-x}(OH)_y$ and $Bi_zIn_{2-z}O_{3-x}(OH)_y$ samples

Sample	Bi to In Molar ratio [a]	BET surface area (m^2 g^{-1})	Eg (eV)	(OH+Ov)/Oxide (Atomic %)[b]	CO_2 capture (mmol g^{-1})	CO Rate (μmol g_{cat}^{-1} h^{-1})	CO Rate Surface area normalized (nmol m^{-2} h^{-1})
Bi-0%	0%	131	2.97	0.83	0.18	0.62±0.04	4.7±0.3
Bi-0.01%	0.033%	131	2.90	0.83	0.17	1.01±0.08	7.7±0.6
Bi-0.03%	0.051%	127	2.96	0.75	0.17	1.32±0.07	10.4±0.6
Bi-0.05%	0.068%	129	2.91	0.63	0.18	1.14±0.06	8.8±0.5
Bi-0.08%	0.114%	132	2.89	0.49	0.14	1.16±0.05	8.8±0.4
Bi-0.1%	0.140%	129	2.86	0.44	0.19	1.17±0.05	9.1±0.4
Bi-0.5%	0.684%	132	2.37	0.64	0.16	0.32±0.1	2.4±0.8
